# Association of COVID 19 pneumonitis and acute mesenteric ischemia

**DOI:** 10.1002/ccr3.8225

**Published:** 2024-02-15

**Authors:** James Norman, Mohamed Siddig Mohamed, Hussam Khougali Mohamed, Mirza Khurrum Baig

**Affiliations:** ^1^ Department of General Surgery University Hospitals Sussex Sussex UK; ^2^ Faculty of Medicine University of Gezira Wad Madani Sudan

**Keywords:** COVID 19, enterocutaneous fistula, mesenteric ischemia, mesenteric thrombosis

## Abstract

**Key Clinical Message:**

D. dimer could be useful as an indicator in diagnosis of mesenteric ischemia in COVID patients. A two staged damage control emergency laparotomy is of good benefits in such patients.

**Abstract:**

Bowel ischemia in COVID 19 patients is extremely rare condition results from migrating thrombus formed by a hypercoagulable inflammatory state that is frequently associated COVID 19 infection. A two staged damage control emergency laparotomy is of good benefits in general especially in those with active COVID 19 infection.

## Graphical abstract

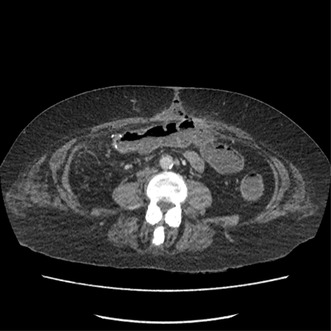

## 1 BACKGROUND

Coronavirus disease (COVID‐19) is an infectious disease caused by the SARS‐CoV‐2 virus discovered in 2019; it is a highly contagious disease and can cause severe acute respiratory distress syndrome as well as other serious conditions.^1^ Within a few months after first being discovered in China, SARS‐CoV‐2 had spread throughout the world, reaching a pandemic level, and causing an international health crisis.^2^ The mortality rate is only 2%, lower than that of strains of SARS discovered in Middle East in the past (9%).^3^, ^4^, ^5^ Patients with COVID‐19 typically appear with respiratory symptoms such fever, coughing, and shortness of breath, however extrapulmonary presentation is uncommon but should not be disregarded.^4^, ^5^ Although findings from the literature have demonstrated a substantial link between COVID 19 and pancreatitis, inflammatory bowel disorders, and intestinal ischemia, acute abdominal manifestations are rare clinical signs in COVID 19 patients.^6^ Bowel ischemia in COVID 19 patients is thought to result from migrating thrombus formed by a hypercoagulable inflammatory state that is frequently associated COVID 19 infection. The virus can also cause progressive disturbance of the homeostatic interactions of coagulation factors, resulting in systemic microvascular thrombi that can migrate and involve intestinal vessels.^7^ The formation of mesenteric artery thrombosis in patients with COVID 19 infection can be very rapid and take place in early stages of the disease.^4^, ^7^ The incidence of Acute Mesenteric Ischemia (AMI), in particular, increased, rising to 1.9%–3.8% in infected individuals.^8^ Clinical intervention depends on early diagnosis; an evaluation regime should be taken into consideration in accordance with the most recent research and recommendations.^7^, ^8^ An accelerated Laparotomies were performed on the majority of patients, with a very significant morbidity and mortality rate.^9^

## 2 CASE PRESENTATION

A 67‐year‐old female patient was admitted to our surgical department complaining of sudden onset intermittent epigastric pain with no fever, vomiting, or change in bowel habits. She had no respiratory symptoms at the time of presentation. She had a history of breast cancer in 2009 which was treated surgically with a wide local excision and completed course of chemotherapy more than 7 years ago. She was very fit and had been well since the surgery.

On clinical examination her abdomen was soft and mildly tender over the epigastric area. Bloods were unremarkable apart from high D‐dimer (Table 1). Vital signs were unremarkable at time of presentation (Table 2). Shortly after admission her O2 saturation deteriorated from 97% to 94% which necessitated support with 2 L of oxygen. She had no recorded spikes temperature and no evidence of COVID 19 symptoms or history of previous infection with COVID 19 was identified (Table 2).

**TABLE 1:** Bloods on admission.

Figure	Patient result	Normal range
Hemoglobin	109	120–150 g/L
White cell counts	17	4–10 × 10*^9^/L
Neutrophils	14.2	2–7 × 10*^9^/L
Lymphocytes	1.7	1–3 × 10*^9^/L
CRP	12	< 1.0
Potassium	5.5	3.5–5.3 mmol/L
D. dimer	2549	0–229 ng/mL
Lactate	1.9	0.5–1.6

**TABLE 2:** Observation on admission.

NEWS	3
BP (blood pressure)	117/95 mmHg
PR (pulse rate)	75 beats/min
RR (respiratory rate)	18 breath/min
O2 sat (oxygen saturation)	95%
Temperature	36.6°C

**FIGURE 1:**
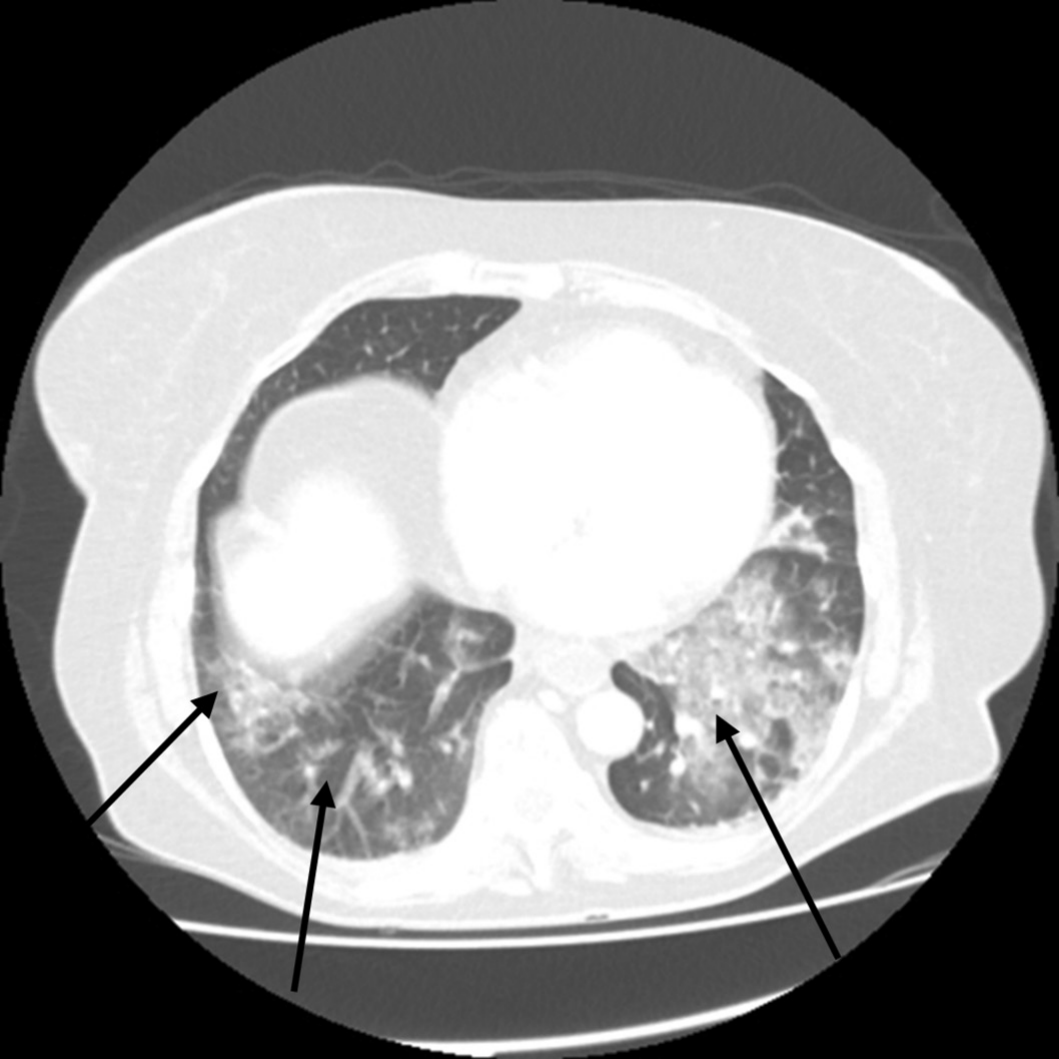
CT scan of the chest; Discrete and confluent ground glass opacities in bilateral visualized lower lobes, concerning for COVID pneumonitis. Minimal bilateral pleural effusion, 26/01/2021.

**FIGURE 2:**
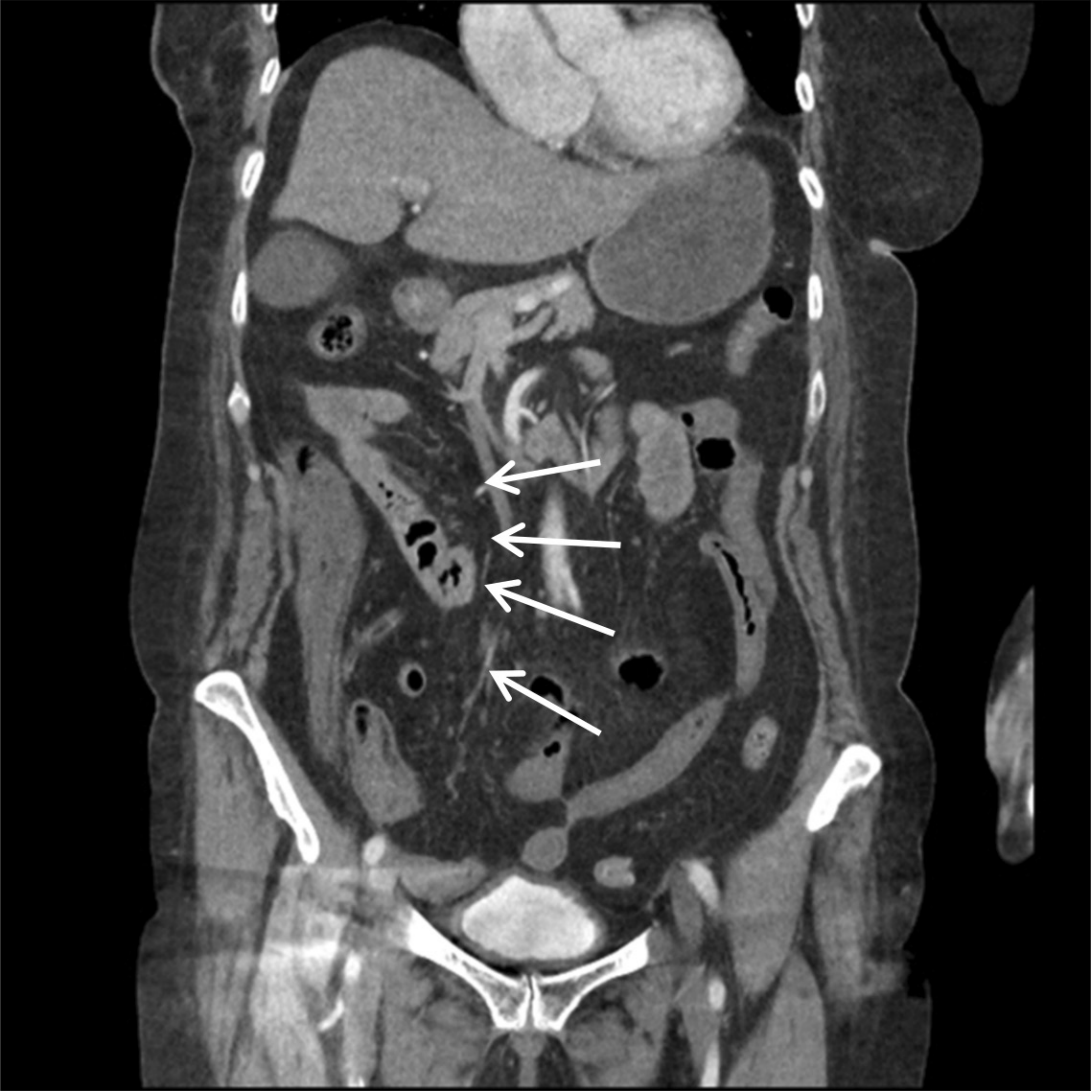
CT angiogram of the abdomen: Thrombus in superior mesenteric artery. Caecum and small bowel loops with apparent poor enhancement which may represent bowel ischaemia. 26/01/2021.

**FIGURE 3:**
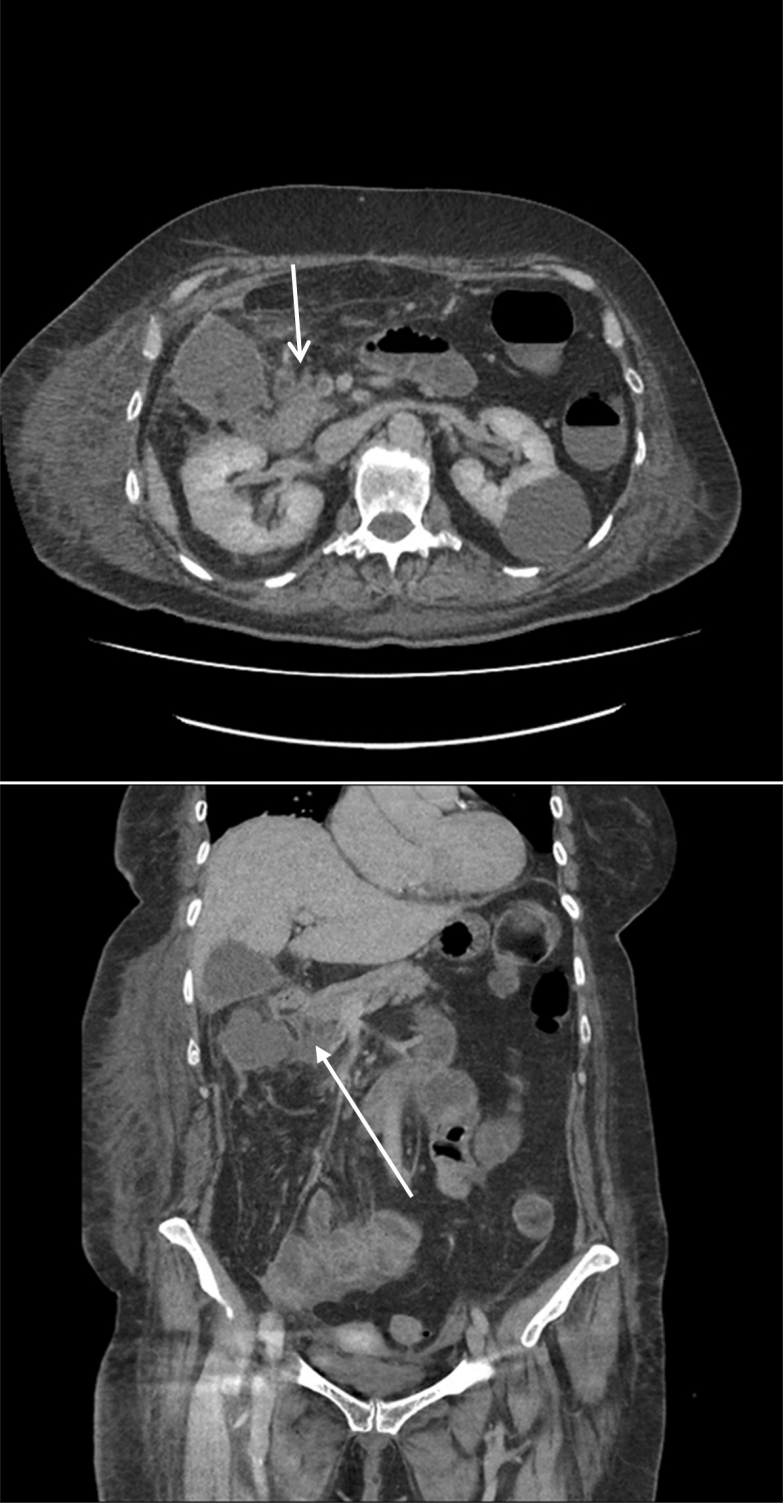
Postoperative CT scan of the abdomen on 12/02/2021: Thrombosed right‐sided tributary of the superior mesenteric vein (SMV).

X‐ray and CT scans of chest on the time of admission showed discrete and confluent ground glass opacities in bilateral visualized lower lobes of the lungs; consistent with COVID 19 pneumonitis. Minimal bilateral pleural effusion was also noted (Figure 1). Specific polymerase chain reaction (PCR) confirmed the diagnosis of COVID‐19 infection. Computerized tomography (CT) angiogram of the abdomen reported low contrast opacification in distal superior mesenteric artery (SMA), which was concerning for SMA occlusion. The caecum and small bowel loops had poor enhancement which was thought to represent bowel ischemia (Figures 2 & 3). This was confirmed when the patient underwent an emergency laparotomy with full COVID 19 pneumonitis. The patient developed intra‐operative atrial fibrillation (heart rate of 122) with fast ventricular response. This required direct current cardioversion to control the heart rate (successful procedure with no cardiac arrest), intra‐operatively.

Intraoperative findings were as following:

1. Extensive small bowel ischemia extended from the jejunum to just proximal to caecum (240 cm)
2. Small Ischaemic patches in the caecum but the remaining colon appeared healthy
3. Free serous fluid in the abdomen

The ischemic small bowel was resected and the remaining viable small bowel length was 60 cm proximally and 10 cm distally. No anastomosis or stoma was formed and a plan for relook surgery after 48 h, treating in the interim with IV heparin, was made. This decision was taken after collective regional consultations from different senior vascular surgeons, consultant general surgeons and anesthetists. A two staged damage control surgery was planned in order to allow time for the patient to recover from the ischemic insult before proceeding with the rest of the surgery and to allow more time for the exact margins of ischemia to be better delineated before further resection.

**FIGURE 4:**
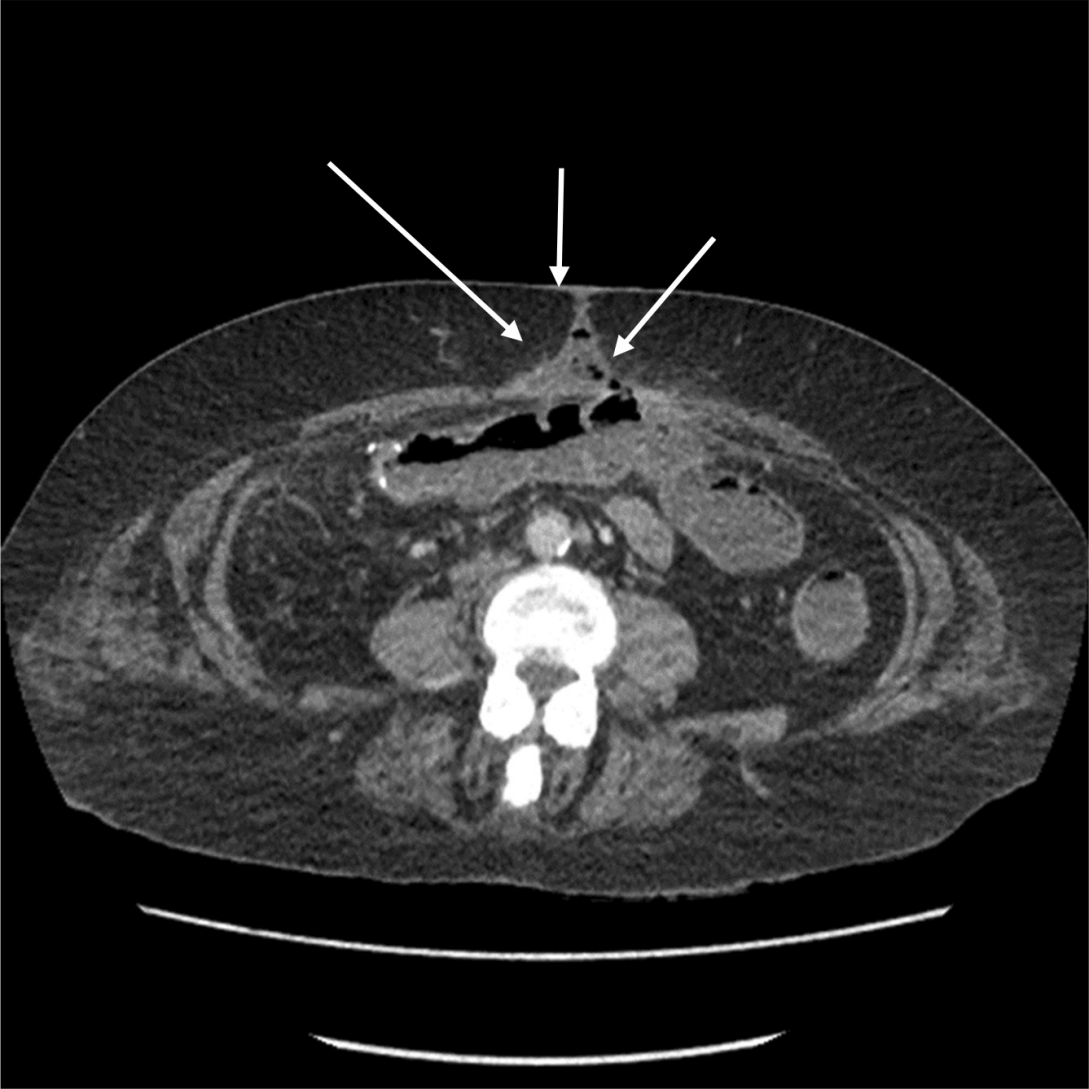
CT scans of the abdomen 20/03/2021; shows New colo‐cutaneous fistula 7.7 cm distal to the anastomosis in the transverse colon.

She subsequently returned to theater and had a right hemicolectomy and a jejuno‐transverse anastomosis as the right colon looked ischemic at the time of re‐inspection. The anastomosis was made due to concerns that she would have long‐term issues with fluid balance if she was left with a high output stoma due to the short length of remaining small bowel. The postoperative phase was eventful with a number of complications as the patient completed 2 months hospital stay (Table 3). Postoperative follows up with CT scans of chest, abdomen and pelvis reported persistent bibasal lung consolidation, bilateral pleural effusion, new onset of thrombus formation in the right sided tributary of the superior mesenteric vein (SMV), wound dehiscence and a colo‐cutaneous fistula (Table 3). (Figure 4). After dramatic hospital events and complications, the patient eventually made a full recovery and was able to resume a respectable quality of life.

**TABLE 3:** postoperative timeline and hospital follow up.

Date	Event
26/01/2021	Laparotomy and Small Bowel resection
28/01/2021	Relook, R hemi colectomy and ileo‐transverse anastomosis
26/01/2021–04/02/2021	ITU admission
01/02/2021	Hypokalemia, Hypernatremia
12/02/2021	Post‐op Intra‐abdominal Collections, Superior mesenteric vein thrombosis and superficial wound dehiscence.
05/03/2021	Bacteremia (Enterococcus durans—unclear source
20/03/2021	New colo‐cutaneous fistula 7.7 cm distal to the anastomosis in the transverse colon.
31/03/2021	Discharged from surgical unit to gastroenterology unit for refeeding support
01/04/2021	Transfer to regional intestinal failure unit for further follow up and rehabilitation

## 3 DISCUSSION

To our knowledge, there was total of eight studies have been published to explore this unusual association: The incidence is greater in men than in women, and it rises sharply with age (all recorded cases, with the exception of two involving women, were men); Ages varied from 10 to 82 years; the median age at diagnosis was around 57 ± 11 years. Our patient is thought to be the first woman to have this uncommon combination of COVID 19 pneumonitis and severe mesenteric ischemia in the small and large bowels to be documented in literature.^10^, ^11^

The average number of days that stomach symptoms last is 4.57 days, ranging from 1 week to 10 days. In about five (62.5%) instances, a high D. dimer level was discovered. Only one (12.5%) incidence of large bowel ischemia was documented, compared to six (75%) cases of extensive mesenteric ischemia of the small intestine. The investigation of choice to identify this issue in each case was a CT scan. One staged laparotomy was preferable surgical option in six (75%) cases while one case (12.5%) treated conservatively (antibiotics, intravenous fluids and anticoagulation). There were three cases survive, two of them treated surgically and one conservatively^7^,^23^ rest of the reported cases died shortly after the operation as results of multi‐organs failure, estimated mortality rate based on available reported cases in literature 5 (62.5%).^12^, ^13^, ^14^

COVID‐19 infection does not involve the lungs only, it can also damage other organs by causing a systematic hyper‐coagulopathy state.^10^ This hyper‐coagulopathy state can result in the development of vascular thromboembolic events, such as pulmonary embolism and deep vein thrombosis (DVT). Therefore, we anticipate that a moving thrombus is what causes intestinal ischemia in COVID 19 individuals. However, it is still unknown what causes this relationship between COVID 19 infection and intestinal ischemia^15^.^16^ Recent research also discovered a strong link between COVID‐19 infection and endothelial damage, which can support the direct production of arterial thrombus.^17^, ^18^

Cheruiyot et al in his review calculated the rates of thrombotic events in systemic arteries occurring in severe/critically ill intensive care unit (ICU)–admitted COVID‐19 patients as 4.4%. Of these the lowest proportion (8%) takes place in the SMA.^19^ SMA thrombosis associated with COVID 19 infection is extremely rare association and hence this can add difficulty for the clinician to identify an early clinical suspicion of this phenomena.

The risk factors of developing bowel ischemia in COVID‐19 patients may include age, obesity, and previous thromboembolic events, cardiac and respiratory comorbidities.^20^ Our patient did not have any of these risk factors before, hence this condition can also be an isolated problem. Elevated D‐dimer levels in asymptomatic COVID‐19 patients could be useful as a risk prediction in newly diagnosed COVID‐19 infection with abdominal pain. Although raised D‐dimer levels are non‐specific, significantly elevated levels are associated with severe disease.

Damage control surgery is a management sequence initiated to reduce the risk of death in severely unwell patients presenting with physiological derangement.^20^ It facilitates a strategy for life‐saving intervention by abbreviated laparotomy with subsequent reoperation for delayed definitive repair after physiological resuscitation. In this scenario, it helped to prevent anesthesia issues related to lengthy surgery. The patient was able to be stabilized in the ICU and had a chance to improve with anticoagulants after having a borderline ischemic region of the colon. This minimized the chance of residual ischaemic bowel or anastomotic failure post operatively. However, the estimated mortality rate in patients with AMI remains high and can be up to 60%.^21^ Additionally, the total morbidity and mortality rate associated with COVID‐19 infection before, during, or after surgery are greater than what would be anticipated and can have a significant impact on postoperative outcomes.^22^, ^23^

Long postoperative hospital stays and development of other complications such as SMV thrombosis, atrial fibrillation, pleural effusions, nutritional deficiency, wound dehiscence, and bowel fistula are all expected in this complex cases. These are not only distressing for patients, stressful for surgeons and can result in worse outcomes, but are also associated with high burden, bed pressures, and increase hospitals costs.

## 4 CONCLUSION

Even if the patient does not exhibit the classic respiratory symptoms of COVID 19, mesenteric ischemia can occur relatively early in patients with new‐onset infections. D. dimer can be employed as a marker for early detection of thrombotic events in COVID patients with additional imaging support, which makes it beneficial in the diagnosis of mesenteric ischemia. For asymptomatic COVID patients who report with abdominal discomfort, an immediate CT angiography of the chest, abdomen, and pelvis is recommended. A two staged emergency laparotomy for damage control has favorable outcomes for patients in general, and perhaps even more so for those who have extensive abdominal pathology in addition to an active COVID 19 infection. Early initiation of thromboprophylaxis in asymptomatic COVID 19 patients can stop thrombosis from developing and contribute to better postoperative patient outcomes.

## AUTHOR CONTRIBUTIONS

**James Norman:** Conceptualization; formal analysis; investigation; project administration; writing – review and editing. **Mohamed Siddig Mohamed:** Formal analysis; methodology; writing – review and editing. **Hussam Khougali Mohamed:** Conceptualization; formal analysis; methodology; writing – review and editing. **Mirza Khurrum Baig:** Formal analysis; supervision; validation.

## CONFLICT OF INTEREST STATEMENT

Authors declare no conflict of interest.

## CONSENT

Written informed consent was obtained from the patient to publish this report in accordance with the journal's patient consent policy.

## Data Availability

The data that support the findings of this study are openly available in PubMed and Google Scholar.
